# Endometrial cancer prognosis prediction using correlation models based on CDK family genes

**DOI:** 10.3389/fgene.2022.1021600

**Published:** 2022-10-10

**Authors:** Xianhua Gu, Honghong Shen, Wenqi Bai, Zheng Xiang, Xinwei Li, Rong Zhang, Fan Shi, Huiyuan Li, Guangzheng Zhu, Suyang Guo

**Affiliations:** ^1^ Department of Gynecological Oncology, First Affiliated Hospital of Bengbu Medical College, Bengbu, China; ^2^ Department of Medical Oncology, First Affiliated Hospital of Bengbu Medical College, Bengbu, China; ^3^ Department of Surgical Oncology, First Affiliated Hospital of Bengbu Medical College, Bengbu, China; ^4^ Department of Pathology, First Affiliated Hospital of Bengbu Medical College, Bengbu, China

**Keywords:** endometrial cancer, cell cycle protein-dependent kinase, CDK16, prognostic model, nomogram

## Abstract

Cyclin-dependent kinases (CDKs) play an important role in cell division. Given that abnormal cell proliferation caused by dysregulation of cell division is one of the major causes of endometrial cancer (EC), it is important to elucidate the role of CDK family genes in the diagnosis and prognosis of EC. In this study, The Cancer Genome Atlas (TCGA) database was used to analyze the frequency of copy number variations and somatic mutations in 26 CDK family genes. Subsequently, the expression of these genes in EC was assessed, and their relationship with overall survival (OS) was examined *via* Kaplan–Meier analysis to assess their prognostic significance. A prognostic model based on seven CDK genes was constructed using Lasso and Cox regression, and the predictive performance of the model was analyzed using Kaplan–Meier analysis and column line plots. The correlation between CDK genes and immune cells was also examined. Patients with EC in the high-risk group had a poorer prognosis. The results of qRT-PCR and immunohistochemical analyses validated that *CDK16* is highly expressed in EC tissues. Patients with EC with high *CDK16* expression had worse 10-year OS than patients with low *CDK16* expression. These findings suggest that the prognostic model constructed based on CDK genes can help to develop individualized and targeted treatment strategies for patients with EC.

## Introduction

Cyclin-dependent kinases (CDKs) are key proteins involved in the cell cycle and transcription. The human genome contains 21 genes encoding CDKs and 5 genes encoding CDK-like (CDKL) proteins (Malumbres et al., 2009). These CDKs function at various stages of the cell cycle and may act as specific protein chaperones that are essential for the functioning of the cell cycle (van den Heuvel, 2005). The importance of CDKs has led to an increase in the use of CDK inhibitors in the treatment of cancer (Malinkova et al., 2015; Goel et al., 2020; Bury et al., 2021), including advanced-stage cancer (Chohan et al., 2018). The role of CDK inhibitors has been extensively investigated in individual cancers such as lung cancer, rectal cancer, breast cancer, pancreatic ductal adenocarcinoma, and gastrointestinal tract cancer (Balakrishnan et al., 2016; Garcia-Reyes et al., 2018; Zhang et al., 2019; Ding et al., 2020; Qin et al., 2020).

Endometrial cancer (EC) is the sixth most common neoplasm among women worldwide (Lortet-Tieulent et al., 2018). In the United States (USA), EC is the most prevalent cancer of the female reproductive system (Siegel et al., 2020). Although the prognosis is usually favorable, high-grade EC has a propensity to recur. Prevention of recurrence is essential because the prognosis of recurrent EC is very poor. At present, traditional and minimally invasive surgeries are the two most crucial treatment options for EC (Chambers et al., 2019; Wright et al., 2019). Recently, there has been an increase in the use of radiotherapy, chemotherapy, targeted therapy, and immunotherapy for treating EC (Ott et al., 2017; Makker et al., 2019; Aoki et al., 2020).

Although CDKs have been extensively investigated in several cancer types, their role in EC remains elusive. In this study, we examined the expression of 26 CDK family genes in healthy endometrial and EC tissues and assessed their relationship with the survival outcomes of patients with EC. Additionally, we constructed a risk score model based on CDK genes and plotted a column line graph for predicting the prognosis of EC.

## Materials and methods

### Endometrial cancer dataset source and preprocessing

Our research flowchart is shown in (Supplementary Figure S1). Clinical data from the TCGA database was obtained data for patients with uterine corpus endometrial carcinoma including total mortality and prognosis, as well as data on common gene expression. The background was corrected and quantile normalization was carried out using multi-array averaging techniques of Affy and simpleaffy. The TCGA Genome Data Commons (GDC, https://portal.gdc.cancer.gov/) was used to download RNA sequencing (FPKM values) and cytogenetic mutation data, which were then thoroughly evaluated using the R package TCGAbiolink (Colaprico et al., 2016). The FPKM values were converted to transcript per kilobase million values. The “ComBat” algorithm of the sva package was used to correct the batch effect of non-biological technical bias. All data were examined using R (version 4.1.2) and the R Bioconductor package.

### Copy number and mutation analysis of cyclin-dependent kinases family genes

The expressions of 26 CDK family genes were retrieved from the TCGA database. Copy number variation (CNV) analysis was performed using Perl software (5.32.1.1) and R (4.1.2), and the distribution of the CDK family genes on the chromosomes was obtained using the RCircos package. The maftools package was used to generate waterfall maps of the mutations. The effect of single mutations on the expression level of other genes was further investigated using Student’s t-test. The ggplot2 package was used to visualize the direction and size of associations between gene expression and mutations.

### Prognostic analysis of cyclin-dependent kinases genes and consensus clustering analysis

The Kaplan–Meier plotter was used to analyze the relationship between CDK family genes and the prognosis of patients with EC. The igraph, psych, reshape2, and RColorBrewer packages in R were used to establish a co-expression network of CDK genes.

### Construction of risk scoring model

The full TCGA set was randomly chosen to serve as both a training dataset and a testing dataset based on the expression profile and survival statistics of CDK family genes. The training dataset was used to construct a CDK family genes model, which was then applied to the complete and test datasets to ensure its accuracy. To evaluate the relationship between pyroptosis-related genes and survival status, the Lasso and Cox regression (“glmnet” and “survival” package) were used. Cross-validation was used to create a reliable model for the Lasso regression. Seven genes were shown to be related to survival based on the penalty parameter (λ), and a multivariate Cox regression model was built using these genes. The optimal set of genes was chosen and used to predict survival using the forward-backward Cox regression algorithm. Survival curves were developed for the training and test datasets using the Kaplan-Meier approach. This formula was used to determine risk scores.

$$\text{Risks }\text{core = }\sum_{\text{i=1}}^{\text{n}}\text{c}\text{o}\text{e}\text{f}\text{i}\;\times \text{Xi}$$

### Comparison of immune cell infiltration

To determine the relative abundance of tumor-infiltrating immune cells (TIICs) in EC samples, the extent of infiltration was estimated using the CIBERSORT algorithm (Chen et al., 2018). A *p*-value of <0.05 indicated significant differences in immune cell infiltration between the two groups.

### Functional analyses of cyclin-dependent kinases family genes in endometrial cancer

The clusterProfiler package was used for Kyoto Encyclopedia of Genes and Genomes (KEGG) and Gene Ontology (GO) enrichment analyses of differentially expressed genes (DEGs). GO analysis considers three aspects for characterizing gene functions, namely, biological processes, cellular components, and molecular functions. Significant DEG-related signaling pathways were mapped on a bubble graph. For gene set enrichment analysis (GSEA), the candidate genes were divided into the risk-high group and the risk-low group, based on the mean of risk score. Functional predefined gene sets were obtained from the Molecular Signatures Database, MSigDB (https://www.gsea-msigdb.org/gsea/msigdb). The candidate genes involved in the pathway with the screening criteria of *p* < 0.05 and false discovery rate (FDR) < 0.25 were considered significantly enriched. The normalized enrichment score and adjusted *p*-value were applied to select the significantly enriched signaling pathways.

### Collection of clinical samples

Tissue specimens were collected from patients with EC undergoing surgery in the Department of Gynecological Oncology of the First Affiliated Hospital of Bengbu Medical College between January and December 2021. A total of 10 EC tissue and adjacent normal tissue samples were used for qRT-PCR, whereas 112 EC tissue samples and 10 adjacent normal tissue samples were used for immunohistochemical (IHC) analysis. The patients neither underwent chemotherapy, radiotherapy, or biological treatment preoperatively or postoperatively nor were previously diagnosed with EC. Until protein extraction, postoperative tissue samples were stored at −80°C.

### Experimental materials

The rabbit anti-human *CDK16* antibody was purchased from CUSABIO (CSB-PA017648ESR2HU) (100 μl, Wuhan, China). A horseradish peroxidase (HRP)-conjugated anti-rabbit antibody was purchased from Jackson ImmunoResearch Inc. (West Grove, PA, United States). Bovine serum albumin was purchased from Sigma-Aldrich (St. Louis, MO, United States). Skimmed milk and Tween-20 were purchased from Sangon Biotech Co., Ltd. (Shanghai, China). The TRIzol reagent was purchased from Thermo Fisher Scientific (United States). The PrimeScriptTM First Strand cDNA Synthesis Kit was purchased from TaKaRa (Tokyo, Japan), and the SYBR Green Real-Time PCR Master Mix was purchased from TOYOBO (Osaka, Japan).

### Immunohistochemical analysis

All tissue samples were fixed in 4% paraformaldehyde, embedded in paraffin, cut into 4-μm-thick sections, and adhered to slides. After deaffinity under different density gradients of xylene, the slides were rehydrated, and antigens were retrieved with citric acid buffer (pH 7.8, 0.1 M) at approximately 82°C for 24 min. Subsequently, the slides were uniformly coated with endogenous peroxidase blocking solution for 15 min at room temperature to prevent peroxidase activity and were incubated with the anti-*CDK16* primary antibody overnight. The following day, the slides were gently washed with PBS, incubated with a biotin-conjugated secondary antibody for 10 min at room temperature, and treated with streptavidin peroxidase for 5 min. Thereafter, the slides were stained with hematoxylin, washed to remove any remaining debris, and air-dried for IHC analysis.

### Quantitative reverse transcription polymerase chain reaction

Total RNA was isolated using the TRIzol reagent. The RevertAid First Strand cDNA Synthesis Kit was used to reverse transcribe the isolated RNA, and the SYBR Green Realtime PCR Master Mix was used to extract the synthesized cDNA. The following primers were used for qRT-PCR: human *CDK16* forward, 5′-TTG​GGC​CGT​TGT​TC-3'; *CDK16* reverse, 5′-GTG​CTC​ACG​GCG​GCT​C-3'; GAPDH forward, 5′-AAGGTGTTCTECTCGGTGAC-3'; GAPDH reverse, 5′-GAG​GGT​AGA​GGA​CTG​AAT​AGT​ACC​TG-3'. GAPDH was used as an internal control. Each sample from each group was tested thrice, and the paired Student’s t-test was used to analyze qRT-PCR data.

### Construction and evaluation of a nomogram for patients with endometrial cancer

A nomogram model was created to predict the OS of patients with EC at 1, 3, and 5 years using prognostic factors based on the findings of the univariate and multivariate Cox regression analyses. The “RMS” package in R software was used for the nomogram analysis. The nomogram was evaluated graphically by drawing calibration curves that contrasted the observed values with the nomogram-predicted probability (using the Kaplan-Meier method). The scatter points of a properly calibrated nomogram prediction model will fall on a 45°diagonal line. The overall predictive power of the nomogram model was also assessed using the Harrell concordance index (C-index). The C-index has a value between 0.5 and 1, and the greater the C-index, the more accurate the prediction. All statistical analyses in this investigation were two-tailed, and the significance threshold was set at 0.05.

### Statistical analysis

The R software (version 4.1.2), Perl software (version 5.32.1.1) and GSEA (version 4.2.3) were used for statistical analysis. A *p*-value of <0.05 was considered significant.

### Ethics statement

The First Affiliated Hospital of Bengbu Medical College’s Ethics Committee examined and approved the investigations involving human subjects (2021) 143. The participants/patients provided written informed consent to take part in this investigation.

## Results

### Cell cycle regulation and expression of cyclin-dependent kinases family genes

**FIGURE 1:**
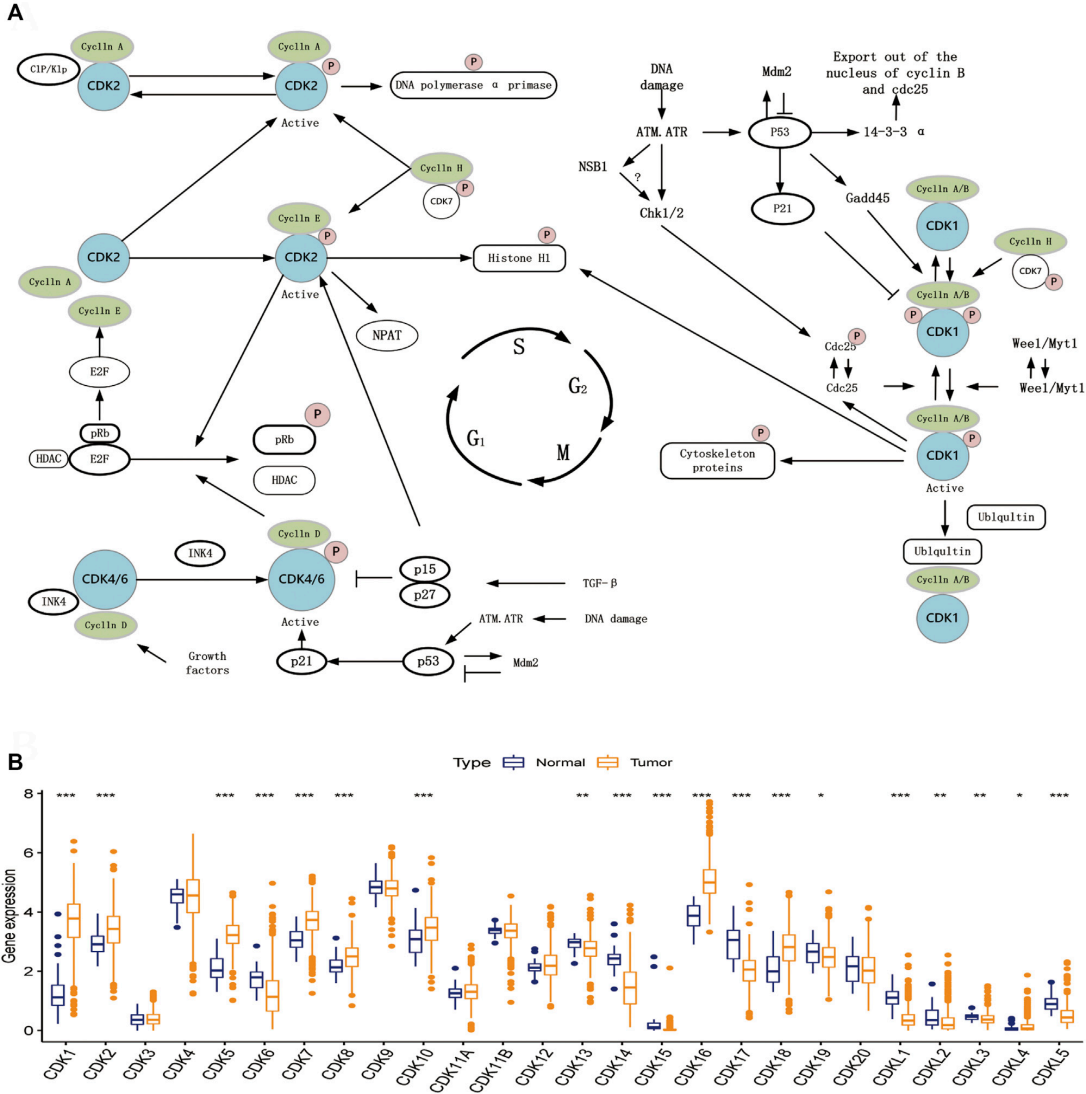
Cell cycle regulation steps and expression of CDK family genes. **(A)** Schematic diagram of some basic steps in cell cycle regulation. **(B)** Expression of 26 CDK family genes in normal endometrial tissue (dark blue) and tumor tissue (dark yellow). Box plots indicate the interquartile range of values. The rows in the boxes indicate median values and the asterisks above indicate *p*-values (*p < 0.05,**p < 0.01,***p < 0.001).

The expression data of 26 CDK family genes were extracted from TCGA database. Figure 1A shows the schematic representation of some basic steps in cell cycle regulation. The mRNA expression of the 26 CDK genes was compared between EC and healthy endometrial tissues to determine whether abnormal expression was associated with EC. Eventually, a total of 19 differentially expressed CDK genes were identified (Figure 1B). These results suggest that the mRNA expression of CDK genes is different between EC and healthy endometrial tissues, and the aberrant expression of CDK genes may be associated with the carcinogenesis and progression of EC.

### Landscape of genetic variations of cyclin-dependent kinases family genes in endometrial cancer

**FIGURE 2:**
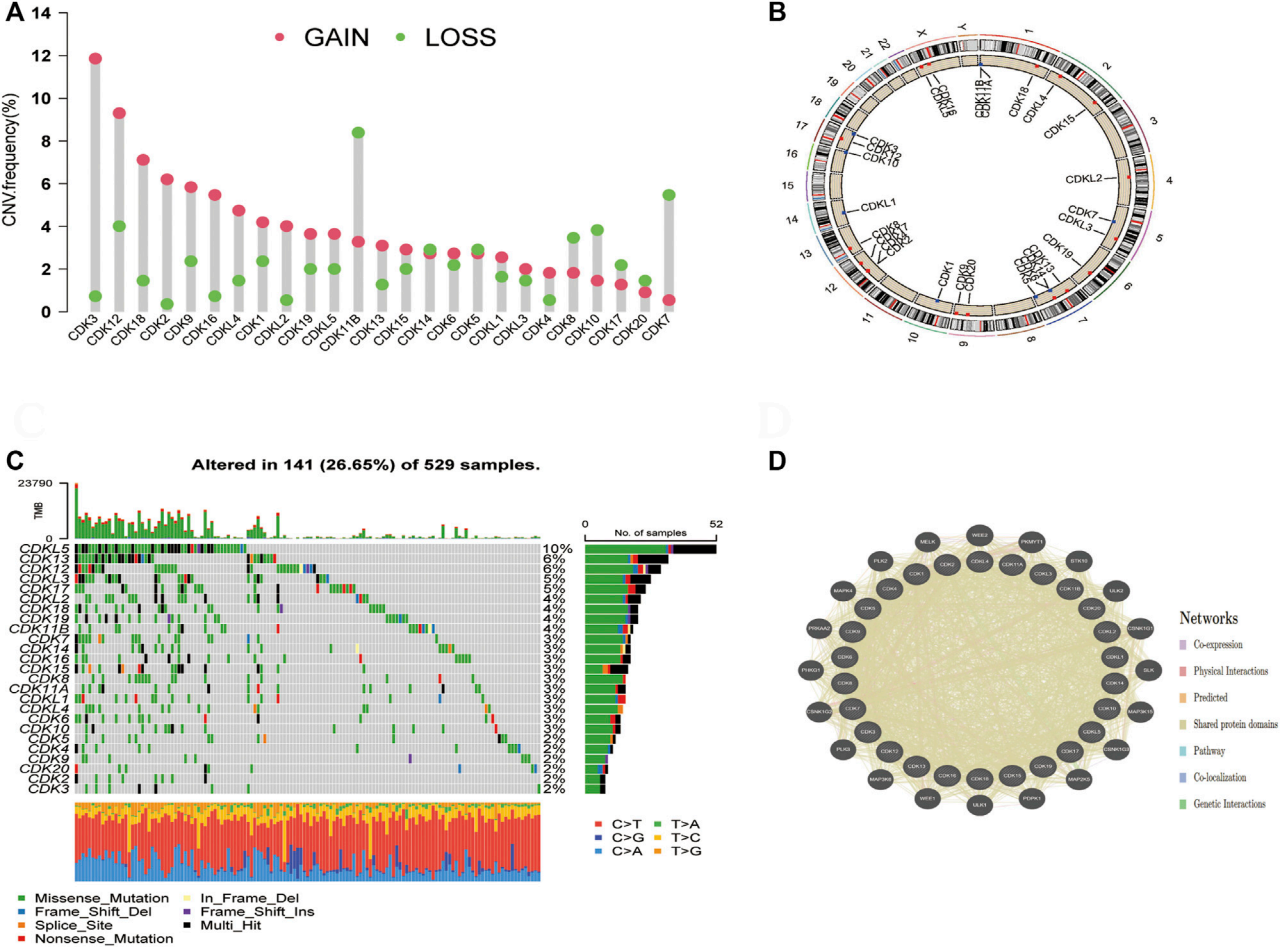
Molecular characterisation and genetic variation of CDK family genes in EC. **(A)** CNV frequencies of CDK genes in EC. The green dots represent the frequency of copy number deletion, the red dots represent the frequency of copy number amplification, and the height of the columns represents a change frequency. **(B)** Location of the CNVs of CDK genes on 23 chromosomes. **(C)** Mutation frequencies of CDK genes in 529 patients with EC TCGA database. Each column represents one patient, the bar on the top represents TMB, and the numbers on the right represent the mutation frequency of each CDK gene. The bar on the right represents the proportion of each CDK gene. The stacked bars below represent the proportion of conversation in each sample. **(D)** Functional interaction network of CDK genes established using the GeneMANIA database.

Copy number variations (CNVs) were prevalent in the 26 CDK genes, especially copy number amplification, whereas the frequency of copy number deletion was high in *CDK7*, *CDK8*, *CDK10*, and *CDK11B* (Figure 2A, Supplementary Table S1). The location of CNVs in CDK genes on chromosomes is shown in Figure 2B. The functional interaction network constructed using the GeneMANIA database showed that genes such as MELK, WEE2, and PKMYT1 were most likely to interact with CDK genes (Figure 2D). Additionally, the frequency of somatic mutations in CDK genes was 26.65% (141/529 samples), and all CDK genes were found to be mutated (Figure 2C).

### Prognostic significance of cyclin-dependent kinases family genes

**FIGURE 3:**
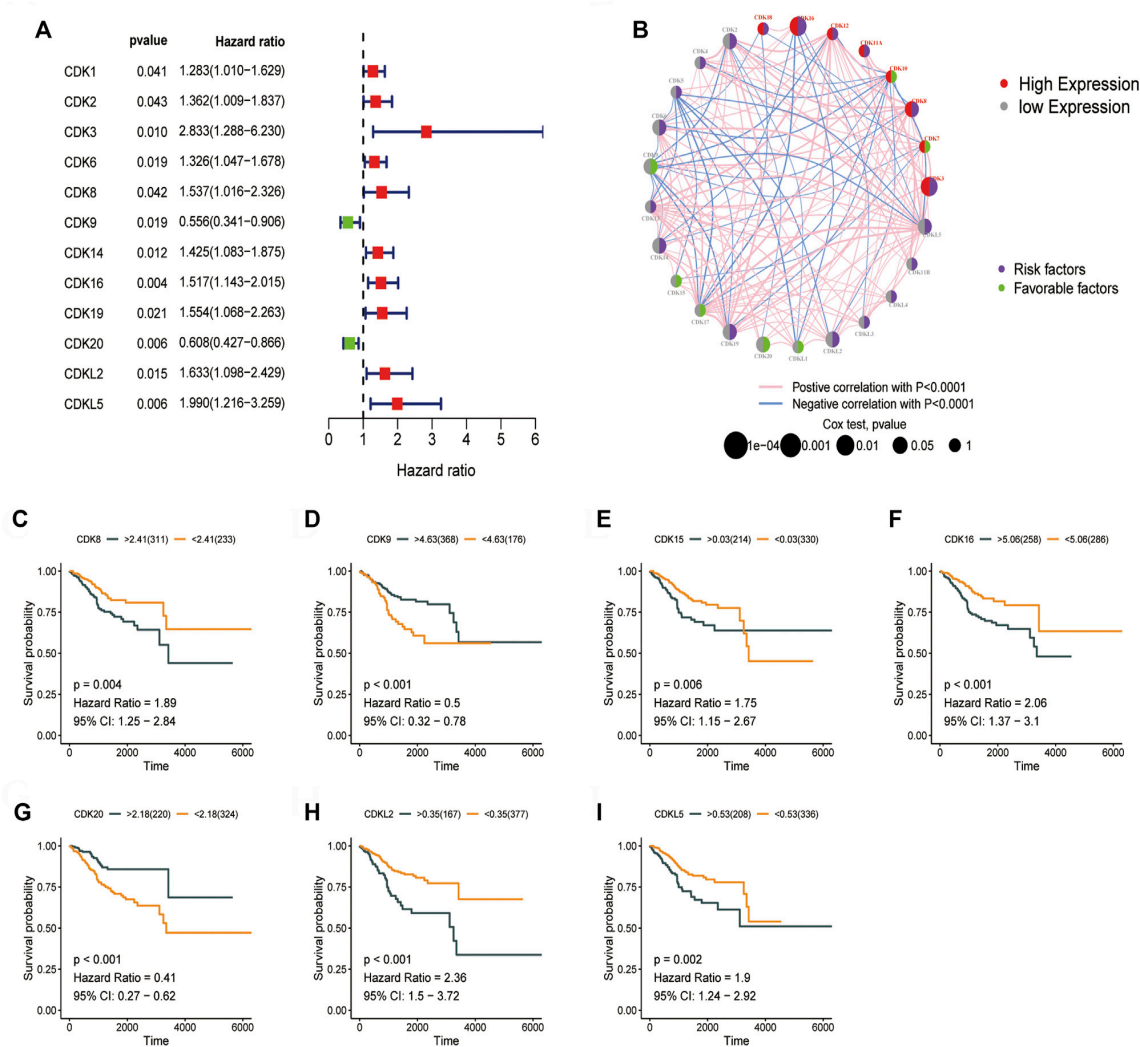
Prognostic significance of CDK family genes. **(A)** Univariate Cox regression analysis was used to analyze CDK family genes. **(B)** Circos plot for univariate cox regression analysis showing the relationship between the prognosis of EC and high (>5, red) and low (<5, grey) expression of CDK genes in TCGA-UCEC dataset (purple, risk factors; green, favorable factors). The *p*-values in Cox regression analysis ranged from 1e-04 to 1 (the larger the bubble, the higher the correlation). **(C–I)** Survival curve of the impact of CDK8, CDK9, CDK15, CDK20, CDKL2, and CDKL5 on OS in TCGA-UCEC dataset.

The prognostic significance of CDK family genes in EC was evaluated. A total of 12 prognostic genes were identified *via* univariate Cox proportional risk regression analysis. Hazard ratios were calculated, and forest plots were generated (Figure 3A). The combined profile of CDK gene interactions, expression, and prognostic significance in EC is shown in Figure 3B. Upregulation or downregulation of most CDK genes had a significant impact on prognosis, and a majority of these genes were identified as risk factors. The relationship between OS and CDK genes was analyzed using the Kaplan–Meier plotter (Figures 3C–I), and patients with low expression of *CDK8* and *CDK16* had better OS.

### Risk score model based on cyclin-dependent kinases family genes

**FIGURE 4:**
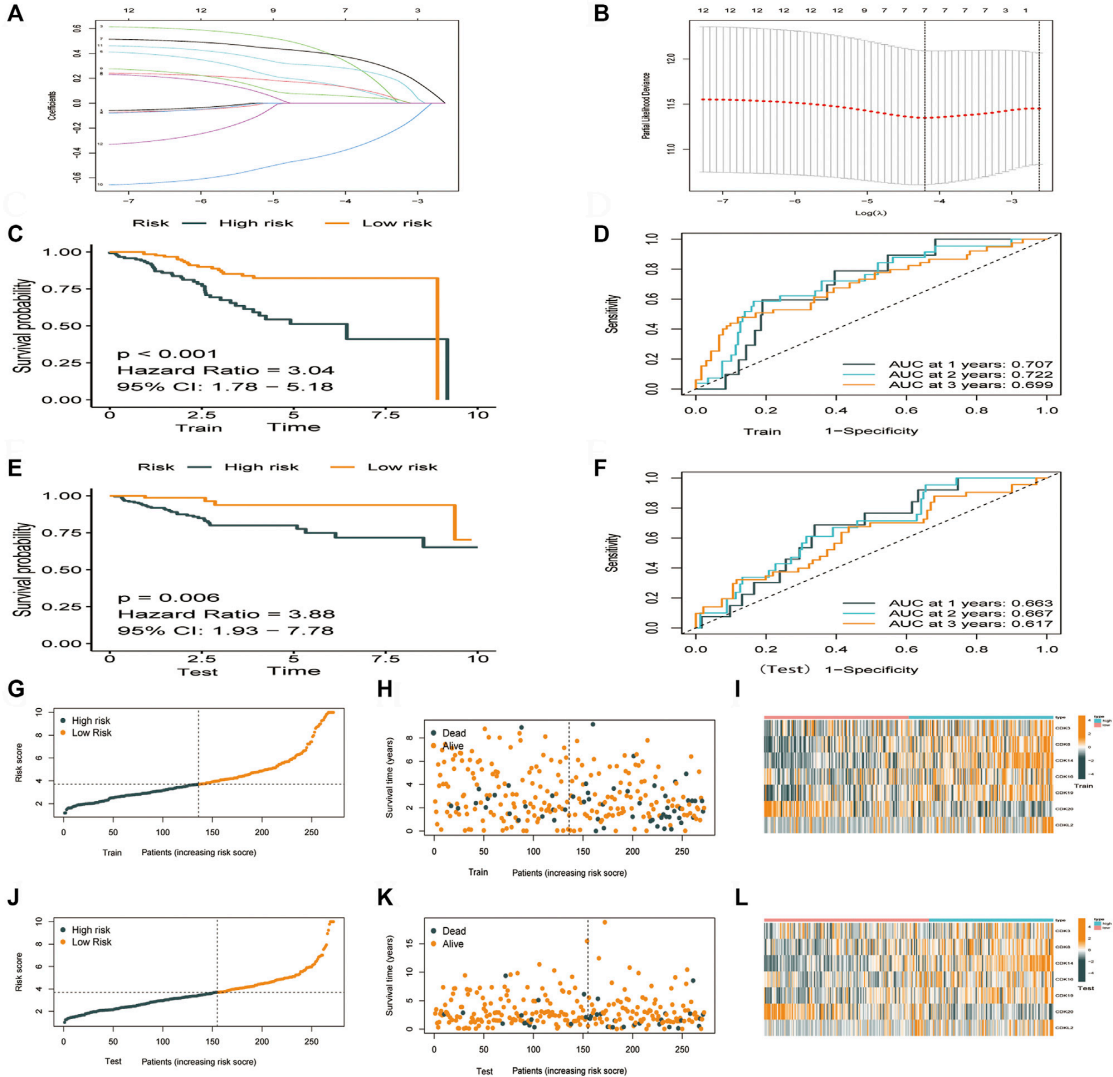
Construction of a prognostic model based on CDK family genes. **(A)** Lasso coefficients of 12 genes associated with OS. **(B)** 10-fold cross-validation error (the first vertical line represents the minimum error, whereas the second vertical line represents the cross-validated error within 1 standard error of the minimum). **(C–L)**. Performance of the risk score model based on seven CDK genes in the training and validation groups. **(C,E)** Kaplan-Meier curves showed that patients with high risk scores had worse OS than patients with low risk score. **(D,F)** ROC curves for predicting survival at 1,3, and 5 years **(G–K)** Survival was longer and mortality was lower in the low-risk group than in the high-risk. **(I,L)** Heatmaps demonstrating the distribution of risk scores in the training **(I)** and validation **(L)** groups.

TCGA-UCEC cohort (544 patients) was divided into the training 272) and validation 272) groups. Lasso–Cox regression analysis was performed to evaluate coefficients for a few selected CDK genes in the training group, the risk score model was developed. The risk score was calculated using the following formula: (0.844 * *CDK3* exp.) + (0.138 * *CDK8* exp.) + (0.280 * *CDK14* exp.) + (-0.365 * *CDK16* exp.) + (0.064 * *CDK19* exp.) + (-0.365*CDK20 exp.) + (0.142 * *CDKL2* exp.) (Figures 3A,B, Supplementary Tables S2, S3). The training and validation groups were further divided into the high- and low-risk groups based on the median risk score. In the training group, the death rate was 40.2% and 14.2% in the high- and low-risk groups, respectively. In the validation group, the death rate was 23.1% and 9.92% in the high- and low-risk groups, respectively. According to survival analysis, patients in the low-risk group had significantly longer OS than patients in the high-risk group (Figures 4C,E, *p* < 0.001). Furthermore, ROC curves were plotted and the area under the curve (AUC) was estimated to assess the accuracy of the risk model in predicting survival (Figures 4D,F). Heatmaps were plotted to demonstrate the survival status of seven independent prognostic genes, risk score distributions, and expression differences were used to evaluate the risk model constructed using the TCGA database (Figures 4G–L). The prognostic model demonstrated excellent prognostic prediction ability, indicating that it can accurately predict the onset and development of EC.

### Construction and assessment of nomogram

**FIGURE 5:**
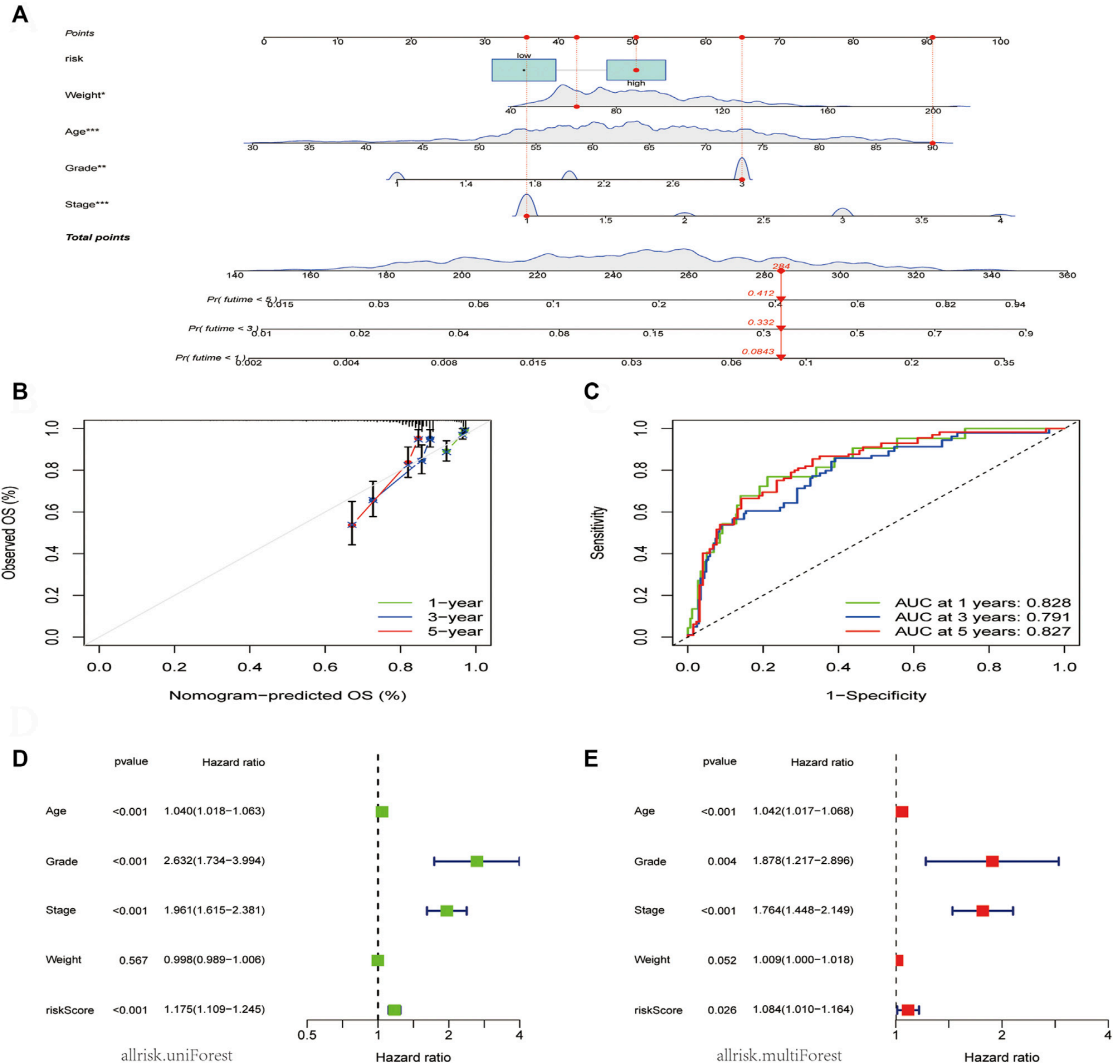
Nomogram model construction and prognostic factor analysis. **(A)**. A nomogram model was constructed to predict the 1-year, 3-year, and 5-years OS probabilities of patients with EC. **(B)** Calibration curves for the nomogram model to predict the probability of the 1-year, 3-year, and 5-year OS of patients with EC. **(C)** ROC curves for the nomogram model to predict the probability of the year 1-year, 3-year, and 5-year OS patients with DC. **(D,E)** The results of univariate **(D)** and multivariate Cox regression **(E)** for the OS of patients with EC are shown in forest plots.

To increase the clinical utility and usability of the CDK-based risk signature, a nomogram was constructed (Figure 5A). Each patient was assigned a total point value by adding the point for each prognostic parameter. The clinical outcome of patients was worse when the total points were higher. The calibration curve showed that the performance of the nomogram was comparable to that of an ideal model (Figure 5B). Additionally, the receiver operating characteristic receiver operating characteristic (ROC) curve showed that the nomogram had a good survival status prediction capacity and accuracy (Figure 5C). Cox regression analysis showed that the CDK family gene prognostic signature and the age, grade, and stage were associated with the prognosis of patients with EC.

The independent significance of the CDK family gene prognostic signature was assessed by examining the model and the clinical prognostic parameters across the entire dataset using univariate and multivariate Cox regression. Age, grade, stage, and weight made up the clinical prognostic parameters. The results of the univariate Cox regression analysis showed that the CDK family gene prognostic signature and the age, grade, and stage were associated with the prognosis of patients with EC (*p* < 0.05) (Figure 5D). Meanwhile, the multivariate Cox regression analysis showed that the CDK family gene prognostic signature, age, grade, and stage were independent prognostic factors for patients with EC, whereas the weight type was not (Figure 5E).

### Hub genes are significantly related to immune cell infiltration

**FIGURE 6:**
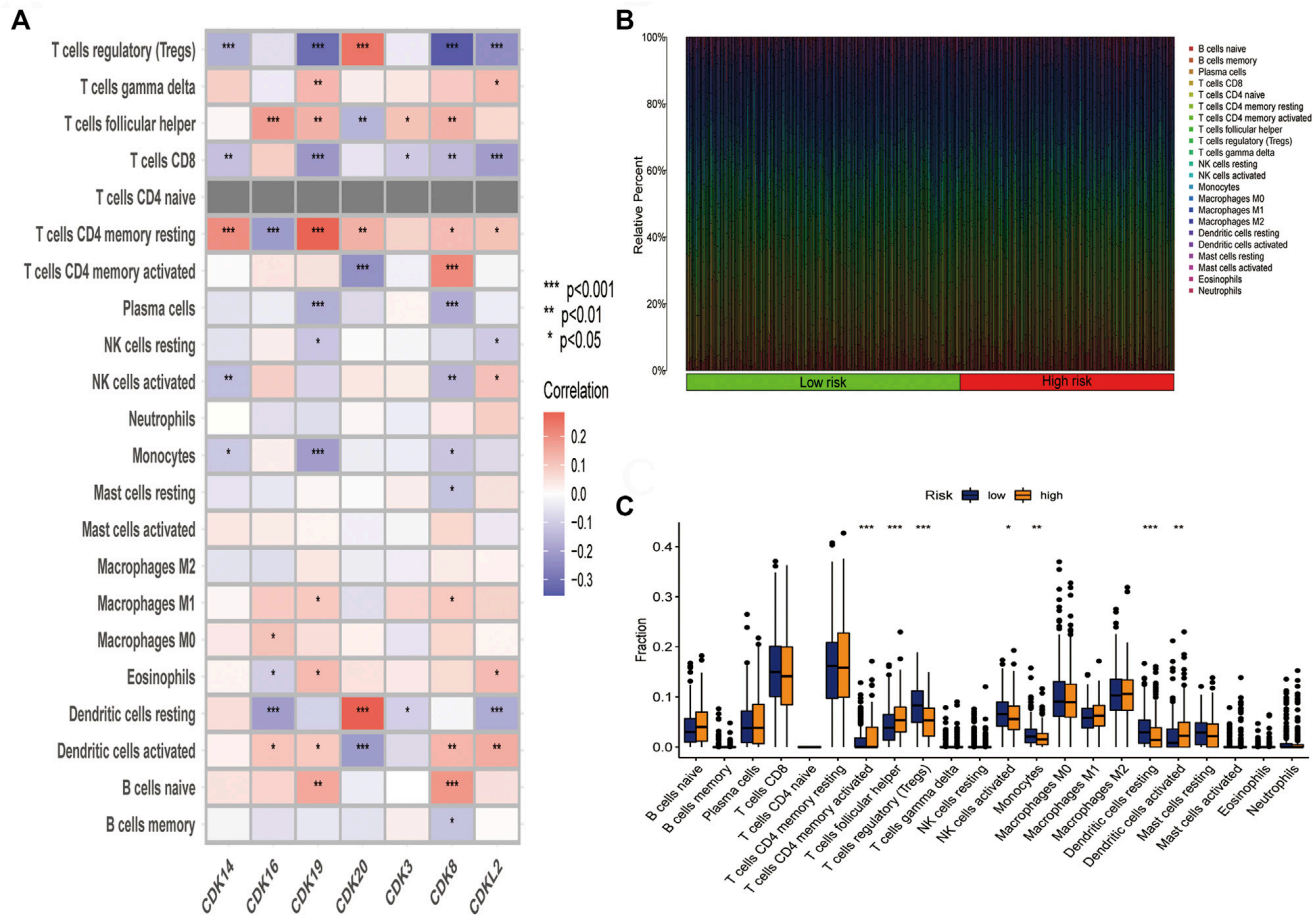
The 7 core prognostic genes were significantly associated with immune cell infiltration. **(A)** Correlation between the 7 prognostic genes and 22 types of infiltration immune cells. **(B)** Histogram demonstrating the difference in the proportion of 22 types of minimum cells in the high-and low-risk groups. **(C)** Box plots demonstrating the difference in the proportion of 22 types of immune cells in the high- and low risk groups.

To examine the correlation between the risk model and immune cell infiltration, the relationship between the 7 core prognostic genes and 22 types of TIICs was examined using the CIBERSORT algorithm (Figure 6A; Supplementary Table S4). The results revealed that *CDK8* was negatively correlated with regulatory T cells and plasma cells and positively correlated with activated memory CD4 T cells and naive B cells. In addition, *CDK16* was negatively correlated with resting dendritic cells and resting memory CD4 T cells and positively correlated with follicular helper T cells and M0 macrophages. A histogram was plotted to demonstrate the levels of immune cell infiltration in the high-and low-risk groups (Figure 6B). Furthermore, the proportion of immune cells was compared between the high- and low-risk groups (Figure 6C). The proportion of regulatory T cells, activated NK cells, and monocytes was significantly higher in the low-risk group, whereas that of follicular helper T cells, activated dendritic cells, and activated memory CD4 T cells was significantly higher in the high-risk group.

### Functional analyses of cyclin-dependent kinases family genes in endometrial cancer

**FIGURE 7:**
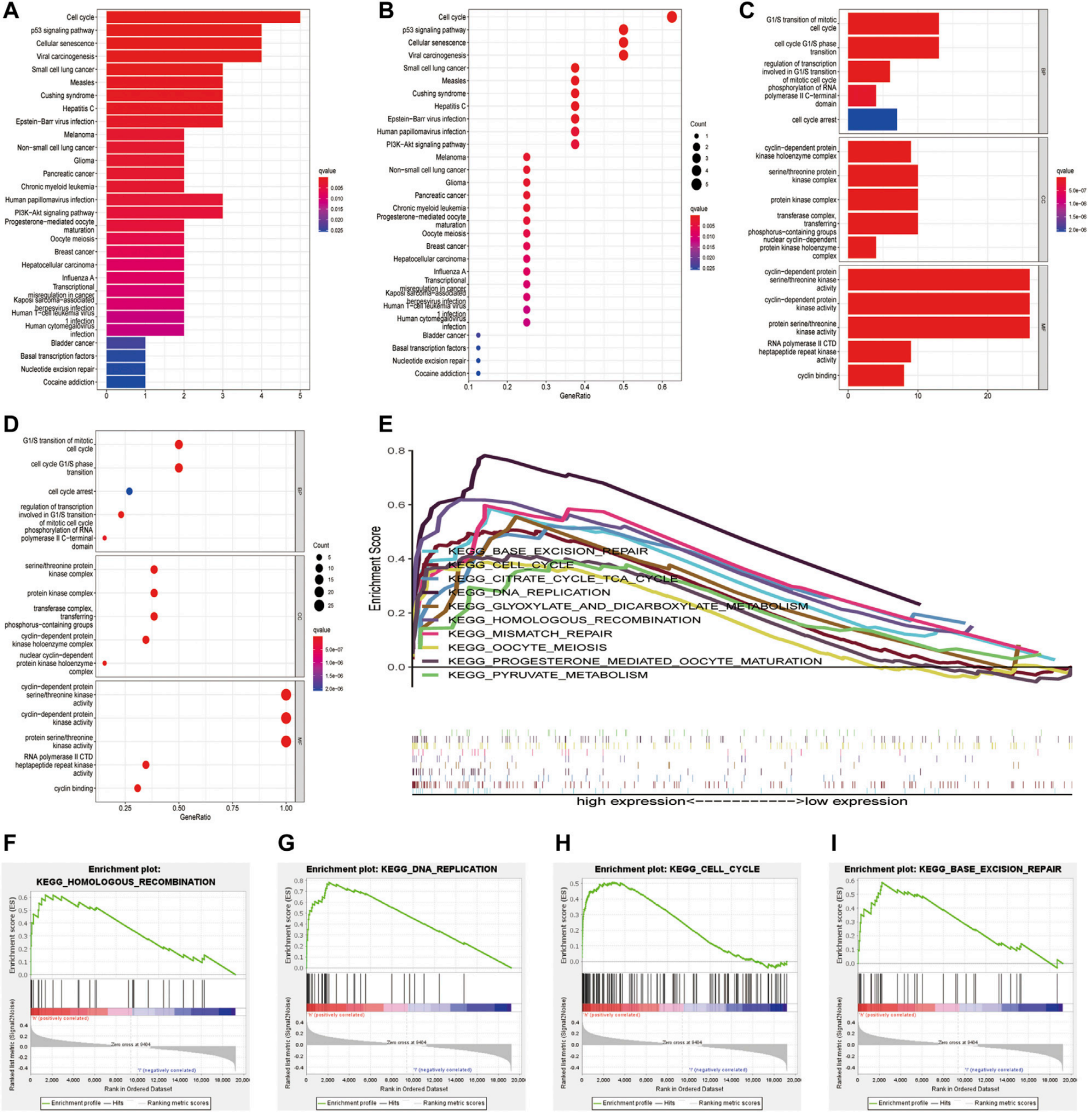
Functional enrichment analysis of CDK family genes in endometrial cancer. **(A,B)** KEGG enrichment analysis of CDK genes. **(C,D)** GO functional annotation of CDK genes. **(E–I)** Significant pathways in the high-and low-risk groups were analysed *via* GSEA.

The clusterProfiler package was used for GO and KEGG functional enrichment analyses of DEGs (Figures 7A–D). KEGG analysis showed that the DEGs were enriched in the cell cycle, p53 signaling pathway, cellular senescence and viral carcinogenesis (Figures 7A,B). GO analysis showed that the DEGs were enriched in biological processes such as the G1/S-phase transition of the mitotic cell cycle; cellular components such as the serine/threonine protein kinase complex, protein kinase complex, and transferase complex; and molecular functions such as cyclin-dependent protein serine/threonine kinase activity and cyclin-dependent protein kinase activity (Figures 7C,D). These findings indicate that CDK genes may play a key role in cell cycle-related signaling pathways. Furthermore, GSEA was used to examine the top 10 relevant signaling pathways in the high-risk group (Figure 7E). The results showed that DEGs in the high-risk group were significantly enriched in pathways associated with functional cell structure and cell cycle, including homologous recombination, DNA replication, and base excision repair (Figures 7E–I). Therefore, the seven core prognostic genes may play an important role in cellular functional architecture and cell cycle, thus promoting the development and progression of EC.

### Expression of *CDK16* in endometrial cancer and adjacent normal tissues

**FIGURE 8:**
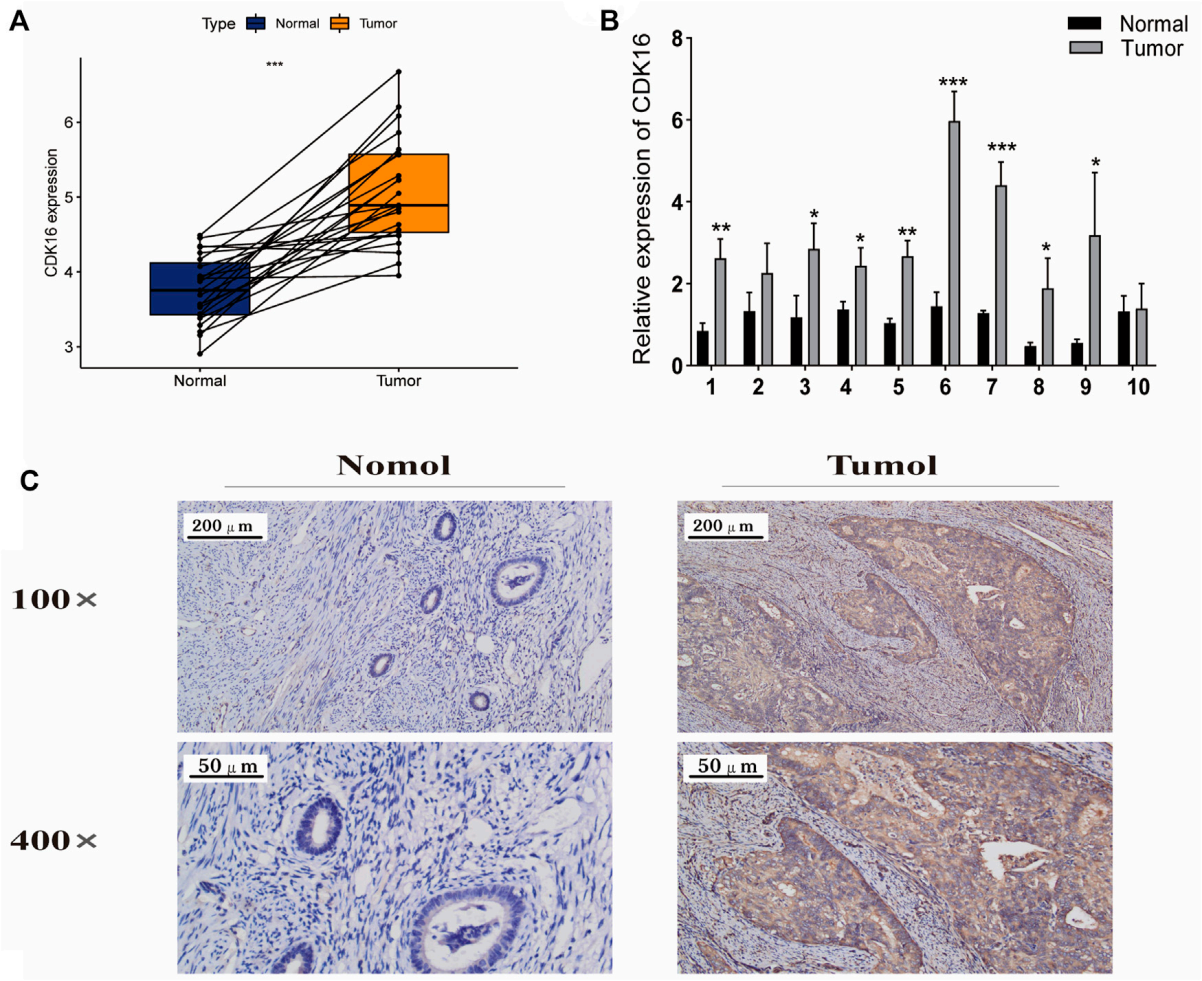
Expression of CDK16 in EC and adjacent normal tissues. **(A)** CDK16 expression was higher in tumour tissues than in adjacent normal tissues in TCGA cohort (*p* < 0.001). **(B)** qRT-PCR revealed that CDK16 expression was higher in tumour tissues than in adjacent normal tissues. **(C)** Immunohistochemical analysis revealed that CDK16 expression was higher in tumour than in adjacent normal tissues (*,*p* < 0.005; **p < 0.01; ***p < 0.001).

Next, we further analyzed the 7 core prognostic genes. In Figure 1B we found higher expression of *CDK8* and *CDK16* in EC tissues than in adjacent normal tissues (*p* < 0.05), while *CDK14*, *CDK15,* and *CDK19* were lower expressed in EC tissues than in adjacent normal tissues. The expression of *CDK3* and *CDK20* in EC tissues was not statistically significant compared with the expression in adjacent normal tissues (*p* > 0.05). In Figures 3C,F we found that EC patients with high *CDK8* and *CDK16* expression had shorter Overall survival (*p* < 0.05). The results showed that *CDK8* and *CDK16* have the potential to be prognostic biomarkers for EC and therefore we are more interested in *CDK8* and *CDK16*. In previous studies, researchers have used *in vitro* experiments to demonstrate that *CDK8* expression is higher in EC tissues than in adjacent normal tissues and that *CDK8* acts as a tumor suppressor in EC (Gu et al., 2013). However, no study has examined the role of *CDK16* in EC. Therefore, *CDK16* was the target of interest in this study. The mRNA expression of *CDK16* was higher in EC tissues than in adjacent normal tissues in TCGA-UCEC dataset (*p* < 0.001) (Figure 8A). In addition, the results of qRT-PCR and IHC staining validated that the mRNA expression of *CDK16* was higher in EC tissues than in adjacent normal tissues (*p* < 0.01) (Figures 8B,C). Altogether, these results indicate that *CDK16* is upregulated in EC.

### Correlation between *CDK16* expression and the clinicopathological features of patients with endometrial cancer

**FIGURE 9:**
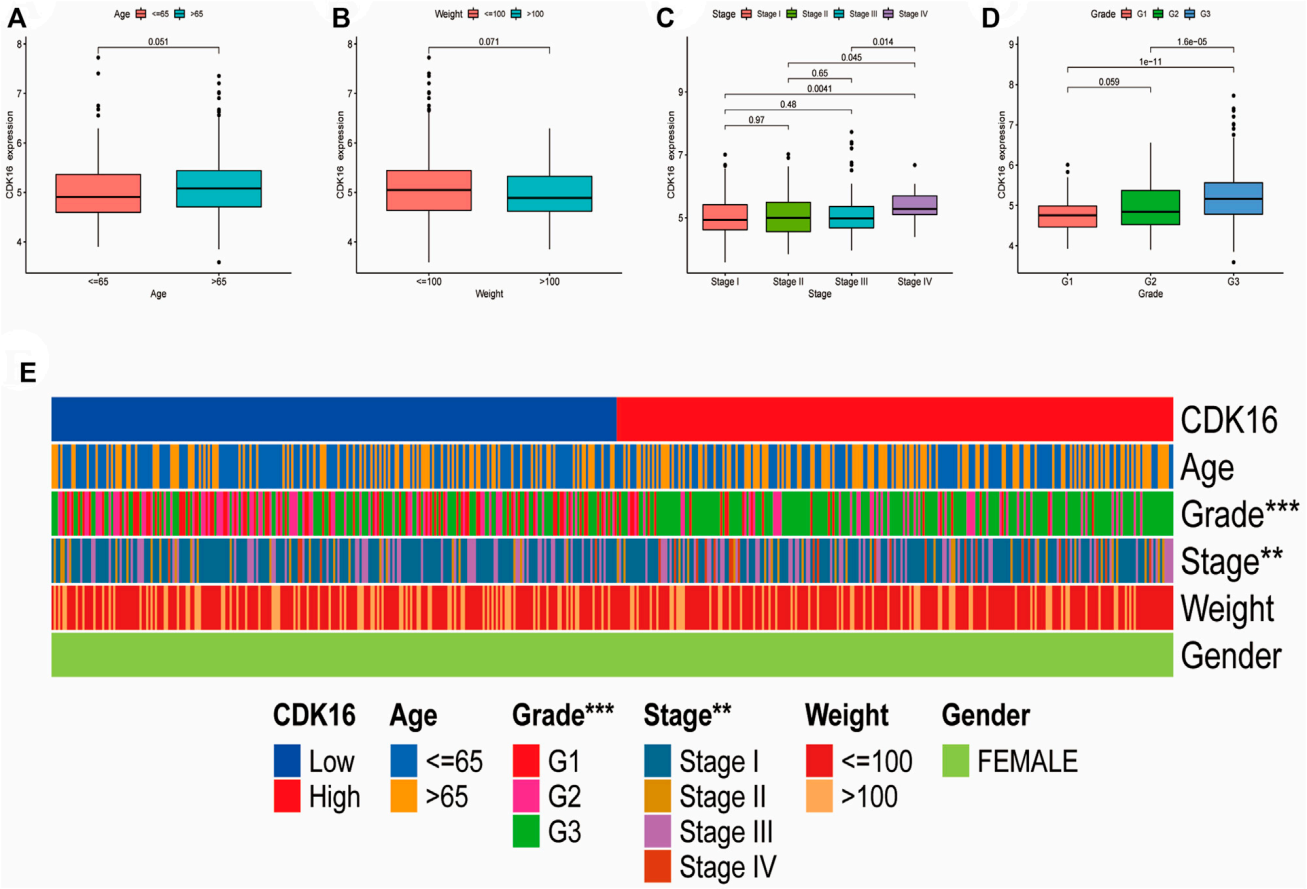
Correlation between CDK16 expression and the clinicopathological features of patients with EC: **(A)** age, **(B)** weight **(C)** pathological stage, **(D)** histological grade. **(E)** Heatmap demonstrating the correlation between CDK16 expression and the clinicopathological characteristics of patients with EC.

Box plots and heatmaps were constructed to demonstrate the correlation between *CDK16* expression and the clinicopathological characteristics of patients with EC (Figures 9A–E). *CDK16* expression was correlated with pathological stage and histological grade (Figures 9C,D) but not with age and weight (Figures 9A,B). These results suggest that *CDK16* plays a key role in the progression of EC.

## Discussion

The human genome has 21 genes that encode CDKs and an additional 5 genes that encode CDKL kinases, a distantly related class of catalytically active proteins that can be controlled by interactions with cell cycle proteins and CDK inhibitors (CKIs) (Malumbres et al., 2009; Lim and Kaldis, 2013). CDK is involved in metabolism, communication, and apoptosis, in addition to transcription and the cell cycle. It also ensures accurate DNA duplication within each cell, resulting in homogeneous DNA duplication within each of the two daughter cells. Additionally, precise gene expression regulation is necessary for healthy growth, whereas transcriptional dysregulation is required for the emergence and spread of cancer (Lukasik et al., 2021; Vervoort et al., 2022). Several studies on CDKs and cancer have been published in recent years, and there is strong evidence that CKIs can be utilized to treat cancer (Leal-Esteban and Fajas, 2020; Jhaveri et al., 2021; Reinius and Smyth, 2021; Liu et al., 2022). There is evidence that targeting CDKs in addition to conventional platinum medications may significantly improve the effectiveness of treatments for ovarian cancer (Zhou, 2017); *CDK4* and *CDK6* inhibitors may be effective in oral squamous cell carcinoma (Kujan et al., 2019); CDK inhibition has the potential to treat pancreatic ductal adenocarcinoma (Garcia-Reyes et al., 2018); breast cancer, including triple-negative and advanced breast cancer, is common cancer for which CKIs are used (Sarosiek, 2018; Quereda et al., 2019; Sofi et al., 2022). The FDA recently approved the dual *CDK4/6* inhibitors Palbociclib, robocalled, and abaculi in combination with other medications for the treatment of hormone receptor positive (HR+) advanced or metastatic breast cancer as well as other breast cancer subtypes (Ding et al., 2020). *CDK7* inhibitors are being investigated as anti-cancer drugs (Sava et al., 2020; Wang et al., 2020; Liang et al., 2021), and *CDK12/13* inhibitors are used in the treatment of a variety of cancers (Tadesse et al., 2021).

*CDK16*, also known as PCTAIRE1 or PCTK1, is a member of the CDK family, which plays an indispensable role in tumorigenesis (Xie et al., 2018). It is closely associated with hepatocellular carcinoma, breast cancer, and non-small cell lung cancer (Wang et al., 2018; Liu et al., 2020; Li et al., 2022). *CDK16* knockdown results in advanced G2 mitotic arrest and abnormal centrosome dynamics in cancer cells, indicating that *CDK16* plays a crucial role in cancer cell proliferation. Therefore, *CDK16* is a desirable target for therapeutic intervention (Yanagi et al., 2016).

Although CKIs have been investigated in EC (Giannone et al., 2019), the results have not been promising. To the best of our knowledge, this study is the first to comprehensively explore the molecular profiles of CDK family genes in EC using TCGA database. The CNV frequency, expression, and prognostic significance of CDK genes in EC were analyzed. Lasso–Cox analysis was used to construct a prognostic model based on seven CDK genes significantly associated with prognosis. Patients with EC were divided into the high- and low-risk groups based on the median risk score, and patients with high-risk scores were found to have a poorer prognosis than those with low-risk scores. Therefore, the risk model exhibited good discriminatory performance in predicting the prognosis of patients with EC. Univariate and multivariate Cox regression analyses showed that the risk score was an independent predictor of prognosis in patients with EC. Additionally, a nomogram was constructed to assess the clinical applicability of the risk model. To assess the relationship between the risk score and immunity, the CIBERSORT algorithm was used to analyze the proportion of TIICs. The proportion of regulatory T cells, activated NK cells, and monocytes was significantly higher in the low-risk group, whereas that of follicular helper T cells, activated dendritic cells, and activated memory CD4 T cells was significantly higher in the high-risk group. These results indicate that the risk score is significantly associated with the immune microenvironment of EC. Regulatory T cells are the internal fighters of the immune system that can counteract pathological immune activation and are associated with immunotherapy (Allos et al., 2019; Akkaya and Shevach, 2020). As powerful effectors of innate immunity, NK cells constitute the first line of defense against cancer (Guillerey, 2020). Monocytes are innate immune cells that play a key role in the development and progression of cancer (Olingy et al., 2019). Dendritic cells are potent antigen-presenting cells associated with the immune response (Brossart et al., 2001). CD4 T cells play an important role in cancer immunology and immunotherapy (Borst et al., 2018). Furthermore, IHC and qRT-PCR analyses revealed that *CDK16* expression was higher in EC tissues than in adjacent normal tissues, and patients with high expression of *CDK8* and *CDK16* were found to have shorter OS.

## Conclusion

In this study, we systematically analyzed the mutation frequency, expression, and prognostic significance of CDK family genes in EC and constructed a risk model based on prognostic CDK genes. Patients with EC were divided into the high- and low-risk groups based on the median risk score. The prognosis of patients in the low-risk group was significantly better than that of patients in the high-risk group. The established nomogram accurately predicted the recurrence of EC and may help to individualize the treatment of EC. The risk score was significantly associated with the immune microenvironment of EC. Additionally, patients with EC with high *CDK16* expression had worse 10-year OS, and *CDK16* expression was correlated with the pathological stage and histological grade of EC. The findings of this study offer valuable insights into developing individualized and targeted therapy for patients with EC.

## Data Availability

The original contributions presented in the study are included in the article/Supplementary Materials, further inquiries can be directed to the corresponding author.

## References

[B1] Akkaya B. Shevach E. M. (2020). Regulatory T cells: Master thieves of the immune system. Cell. Immunol. 355, 104160. 10.1016/j.cellimm.2020.104160 32711171 PMC9761694

[B2] Allos H. Al Dulaijan B. S. Choi J. Azzi J. (2019). Regulatory T cells for more targeted immunosuppressive therapies. Clin. Lab. Med. 39 (1), 1–13. 10.1016/j.cll.2018.11.001 30709499 PMC6501202

[B3] Aoki Y. Kanao H. Wang X. Yunokawa M. Omatsu K. Fusegi A. (2020). Adjuvant treatment of endometrial cancer today. Jpn. J. Clin. Oncol. 50 (7), 753–765. 10.1093/jjco/hyaa071 32463094

[B4] Balakrishnan A. Vyas A. Deshpande K. Vyas D. (2016). Pharmacological cyclin dependent kinase inhibitors: Implications for colorectal cancer. World J. Gastroenterol. 22 (7), 2159–2164. 10.3748/wjg.v22.i7.2159 26900281 PMC4734993

[B5] Borst J. Ahrends T. Babala N. Melief C. J. M. Kastenmuller W. (2018). CD4(+) T cell help in cancer immunology and immunotherapy. Nat. Rev. Immunol. 18 (10), 635–647. 10.1038/s41577-018-0044-0 30057419

[B6] Brossart P. Wirths S. Brugger W. Kanz L. (2001). Dendritic cells in cancer vaccines. Exp. Hematol. 29 (11), 1247–1255. 10.1016/s0301-472x(01)00730-5 11698120

[B7] Bury M. Le Calve B. Ferbeyre G. Blank V. Lessard F. (2021). New insights into CDK regulators: Novel opportunities for cancer therapy. Trends Cell Biol. 31 (5), 331–344. 10.1016/j.tcb.2021.01.010 33676803

[B8] Chambers L. M. Carr C. Freeman L. Jernigan A. M. Michener C. M. (2019). Does surgical platform impact recurrence and survival? A study of utilization of multiport, single-port, and robotic-assisted laparoscopy in endometrial cancer surgery. Am. J. Obstet. Gynecol. 221 (3), e1–e243. e211. 10.1016/j.ajog.2019.04.038 31075245

[B9] Chen B. Khodadoust M. S. Liu C. L. Newman A. M. Alizadeh A. A. (2018). Profiling tumor infiltrating immune cells with CIBERSORT. Methods Mol. Biol. 1711, 243–259. 10.1007/978-1-4939-7493-1_12 29344893 PMC5895181

[B10] Chohan T. A. Qayyum A. Rehman K. Tariq M. Akash M. S. H. (2018). An insight into the emerging role of cyclin-dependent kinase inhibitors as potential therapeutic agents for the treatment of advanced cancers. Biomed. Pharmacother. 107, 1326–1341. 10.1016/j.biopha.2018.08.116 30257348

[B11] Colaprico A. Silva T. C. Olsen C. Garofano L. Cava C. Garolini D. (2016). TCGAbiolinks: An R/bioconductor package for integrative analysis of TCGA data. Nucleic Acids Res. 44 (8), e71. 10.1093/nar/gkv1507 26704973 PMC4856967

[B12] Ding L. Cao J. Lin W. Chen H. Xiong X. Ao H. (2020). The roles of cyclin-dependent kinases in cell-cycle progression and therapeutic strategies in human breast cancer. Int. J. Mol. Sci. 21 (6), E1960. 10.3390/ijms21061960 32183020 PMC7139603

[B13] Garcia-Reyes B. Kretz A. L. Ruff J. P. von Karstedt S. Hillenbrand A. Knippschild U. (2018). The emerging role of cyclin-dependent kinases (CDKs) in pancreatic ductal adenocarcinoma. Int. J. Mol. Sci. 19 (10), E3219. 10.3390/ijms19103219 30340359 PMC6214075

[B14] Giannone G. Tuninetti V. Ghisoni E. Genta S. Scotto G. Mittica G. (2019). Role of cyclin-dependent kinase inhibitors in endometrial cancer. Int. J. Mol. Sci. 20 (9), E2353. 10.3390/ijms20092353 31083638 PMC6539322

[B15] Goel B. Tripathi N. Bhardwaj N. Jain S. K. (2020). Small molecule CDK inhibitors for the therapeutic management of cancer. Curr. Top. Med. Chem. 20 (17), 1535–1563. 10.2174/1568026620666200516152756 32416692

[B16] Gu W. Wang C. Li W. Hsu F. N. Tian L. Zhou J. (2013). Tumor-suppressive effects of CDK8 in endometrial cancer cells. Cell Cycle 12 (6), 987–999. 10.4161/cc.24003 23454913 PMC3637357

[B17] Guillerey C. (2020). NK cells in the tumor microenvironment. Adv. Exp. Med. Biol. 1273, 69–90. 10.1007/978-3-030-49270-0_4 33119876

[B18] Jhaveri K. Burris Rd H. A. Yap T. A. Hamilton E. Rugo H. S. Goldman J. W. (2021). The evolution of cyclin dependent kinase inhibitors in the treatment of cancer. Expert Rev. Anticancer Ther. 21 (10), 1105–1124. 10.1080/14737140.2021.1944109 34176404 PMC12592983

[B19] Kujan O. Huang G. Ravindran A. Vijayan M. Farah C. S. (2019). The role of cyclin-dependent kinases in oral potentially malignant disorders and oral squamous cell carcinoma. J. Oral Pathol. Med. 48 (7), 560–565. 10.1111/jop.12903 31172620

[B20] Leal-Esteban L. C. Fajas L. (2020). Cell cycle regulators in cancer cell metabolism. Biochim. Biophys. Acta. Mol. Basis Dis. 1866 (5), 165715. 10.1016/j.bbadis.2020.165715 32035102

[B21] Li X. Li J. Xu L. Wei W. Cheng A. Zhang L. (2022). CDK16 promotes the progression and metastasis of triple-negative breast cancer by phosphorylating PRC1. J. Exp. Clin. Cancer Res. 41 (1), 149. 10.1186/s13046-022-02362-w 35449080 PMC9027050

[B22] Liang H. Du J. Elhassan R. M. Hou X. Fang H. (2021). Recent progress in development of cyclin-dependent kinase 7 inhibitors for cancer therapy. Expert Opin. Investig. Drugs 30 (1), 61–76. 10.1080/13543784.2021.1850693 33183110

[B23] Lim S. Kaldis P. (2013). Cdks, cyclins and CKIs: Roles beyond cell cycle regulation. Development 140 (15), 3079–3093. 10.1242/dev.091744 23861057

[B24] Liu Q. Wang C. Jiang Z. Li S. Li F. Tan H. B. (2020). circRNA 001306 enhances hepatocellular carcinoma growth by up-regulating CDK16 expression via sponging miR-584-5p. J. Cell. Mol. Med. 24 (24), 14306–14315. 10.1111/jcmm.16047 33135290 PMC7754030

[B25] Liu Y. Fu L. Wu J. Liu M. Wang G. Liu B. (2022). Transcriptional cyclin-dependent kinases: Potential drug targets in cancer therapy. Eur. J. Med. Chem. 229, 114056. 10.1016/j.ejmech.2021.114056 34942431

[B26] Lortet-Tieulent J. Ferlay J. Bray F. Jemal A. (2018). International patterns and trends in endometrial cancer incidence, 1978-2013. J. Natl. Cancer Inst. 110 (4), 354–361. 10.1093/jnci/djx214 29045681

[B27] Lukasik P. Zaluski M. Gutowska I. (2021). Cyclin-dependent kinases (CDK) and their role in diseases development-review. Int. J. Mol. Sci. 22 (6), 2935. 10.3390/ijms22062935 33805800 PMC7998717

[B28] Makker V. Rasco D. Vogelzang N. J. Brose M. S. Cohn A. L. Mier J. (2019). Lenvatinib plus pembrolizumab in patients with advanced endometrial cancer: An interim analysis of a multicentre, open-label, single-arm, phase 2 trial. Lancet. Oncol. 20 (5), 711–718. 10.1016/S1470-2045(19)30020-8 30922731 PMC11686814

[B29] Malinkova V. Vylicil J. Krystof V. (2015). Cyclin-dependent kinase inhibitors for cancer therapy: A patent review (2009 - 2014). Expert Opin. Ther. Pat. 25 (9), 953–970. 10.1517/13543776.2015.1045414 26161698

[B30] Malumbres M. Harlow E. Hunt T. Hunter T. Lahti J. M. Manning G. (2009). Cyclin-dependent kinases: A family portrait. Nat. Cell Biol. 11 (11), 1275–1276. 10.1038/ncb1109-1275 19884882 PMC2914104

[B31] Olingy C. E. Dinh H. Q. Hedrick C. C. (2019). Monocyte heterogeneity and functions in cancer. J. Leukoc. Biol. 106 (2), 309–322. 10.1002/JLB.4RI0818-311R 30776148 PMC6658332

[B32] Ott P. A. Bang Y. J. Berton-Rigaud D. Elez E. Pishvaian M. J. Rugo H. S. (2017). Safety and antitumor activity of pembrolizumab in advanced programmed death ligand 1-positive endometrial cancer: Results from the KEYNOTE-028 study. J. Clin. Oncol. 35 (22), 2535–2541. 10.1200/JCO.2017.72.5952 28489510

[B33] Qin A. Reddy H. G. Weinberg F. D. Kalemkerian G. P. (2020). Cyclin-dependent kinase inhibitors for the treatment of lung cancer. Expert Opin. Pharmacother. 21 (8), 941–952. 10.1080/14656566.2020.1738385 32164461

[B34] Quereda V. Bayle S. Vena F. Frydman S. M. Monastyrskyi A. Roush W. R. (2019). Therapeutic targeting of CDK12/CDK13 in triple-negative breast cancer. Cancer Cell 36 (5), 545–558. 10.1016/j.ccell.2019.09.004 31668947

[B35] Reinius M. A. V. Smyth E. (2021). Anti-cancer therapy with cyclin-dependent kinase inhibitors: Impact and challenges. Expert Rev. Mol. Med. 23, e6. 10.1017/erm.2021.3 34103115

[B36] Sarosiek T. (2018). Inhibitors of cyclin-dependent kinases (CDK) - a new group of medicines in therapy of advanced breast cancer. Pol. Merkur. Lek. 44 (259), 5–9. 29374415

[B37] Sava G. P. Fan H. Coombes R. C. Buluwela L. Ali S. (2020). CDK7 inhibitors as anticancer drugs. Cancer Metastasis Rev. 39 (3), 805–823. 10.1007/s10555-020-09885-8 32385714 PMC7497306

[B38] Siegel R. L. Miller K. D. Jemal A. (2020). Cancer statistics, 2020. Ca. Cancer J. Clin. 70 (1), 7–30. 10.3322/caac.21590 31912902

[B39] Sofi S. Mehraj U. Qayoom H. Aisha S. Asdaq S. M. B. Almilaibary A. (2022). Cyclin-dependent kinases in breast cancer: Expression pattern and therapeutic implications. Med. Oncol. 39 (6), 106. 10.1007/s12032-022-01731-x 35486263

[B40] Tadesse S. Duckett D. R. Monastyrskyi A. (2021). The promise and current status of CDK12/13 inhibition for the treatment of cancer. Future Med. Chem. 13 (2), 117–141. 10.4155/fmc-2020-0240 33295810

[B41] van den Heuvel S. (2005). Cell-cycle regulation. WormBook, 1–16. 10.1895/wormbook.1.28.1 PMC4781127 18050422

[B42] Vervoort S. J. Devlin J. R. Kwiatkowski N. Teng M. Gray N. S. Johnstone R. W. (2022). Targeting transcription cycles in cancer. Nat. Rev. Cancer 22 (1), 5–24. 10.1038/s41568-021-00411-8 34675395

[B43] Wang H. Liu H. Min S. Shen Y. Li W. Chen Y. (2018). CDK16 overexpressed in non-small cell lung cancer and regulates cancer cell growth and apoptosis via a p27-dependent mechanism. Biomed. Pharmacother. 103, 399–405. 10.1016/j.biopha.2018.04.080 29674275

[B44] Wang M. Wang T. Zhang X. Wu X. Jiang S. (2020). Cyclin-dependent kinase 7 inhibitors in cancer therapy. Future Med. Chem. 12 (9), 813–833. 10.4155/fmc-2019-0334 32208930

[B45] Wright J. D. Khoury-Collado F. Melamed A. (2019). Harnessing minimally invasive surgery to improve outcomes in endometrial cancer surgery-the robots are coming. JAMA Surg. 154 (6), 539. 10.1001/jamasurg.2018.5841 30810712

[B46] Xie J. Li Y. Jiang K. Hu K. Zhang S. Dong X. (2018). CDK16 phosphorylates and degrades p53 to promote radioresistance and predicts prognosis in lung cancer. Theranostics 8 (3), 650–662. 10.7150/thno.21963 29344296 PMC5771083

[B47] Yanagi T. Tachikawa K. Wilkie-Grantham R. Hishiki A. Nagai K. Toyonaga E. (2016). Lipid nanoparticle-mediated siRNA transfer against PCTAIRE1/PCTK1/cdk16 inhibits *in vivo* cancer growth. Mol. Ther. Nucleic Acids 5 (6), e327. 10.1038/mtna.2016.40 27351680 PMC5022131

[B48] Zhang J. Su G. Lin Y. Meng W. Lai J. K. L. Qiao L. (2019). Targeting cyclin-dependent kinases in gastrointestinal cancer therapy. Discov. Med. 27 (146), 27–36. 30721649

[B49] Zhou Q. (2017). Targeting cyclin-dependent kinases in ovarian cancer. Cancer Invest. 35 (6), 367–376. 10.1080/07357907.2017.1283508 28406716

