# Supplementary figures and images for: Endometrial cancer prognosis prediction using correlation models based on CDK family genes
Source: Front Genet. 2022 Oct 10;13:1021600. doi: 10.3389/fgene.2022.1021600 (PMC9589062; doi:10.3389/fgene.2022.1021600)

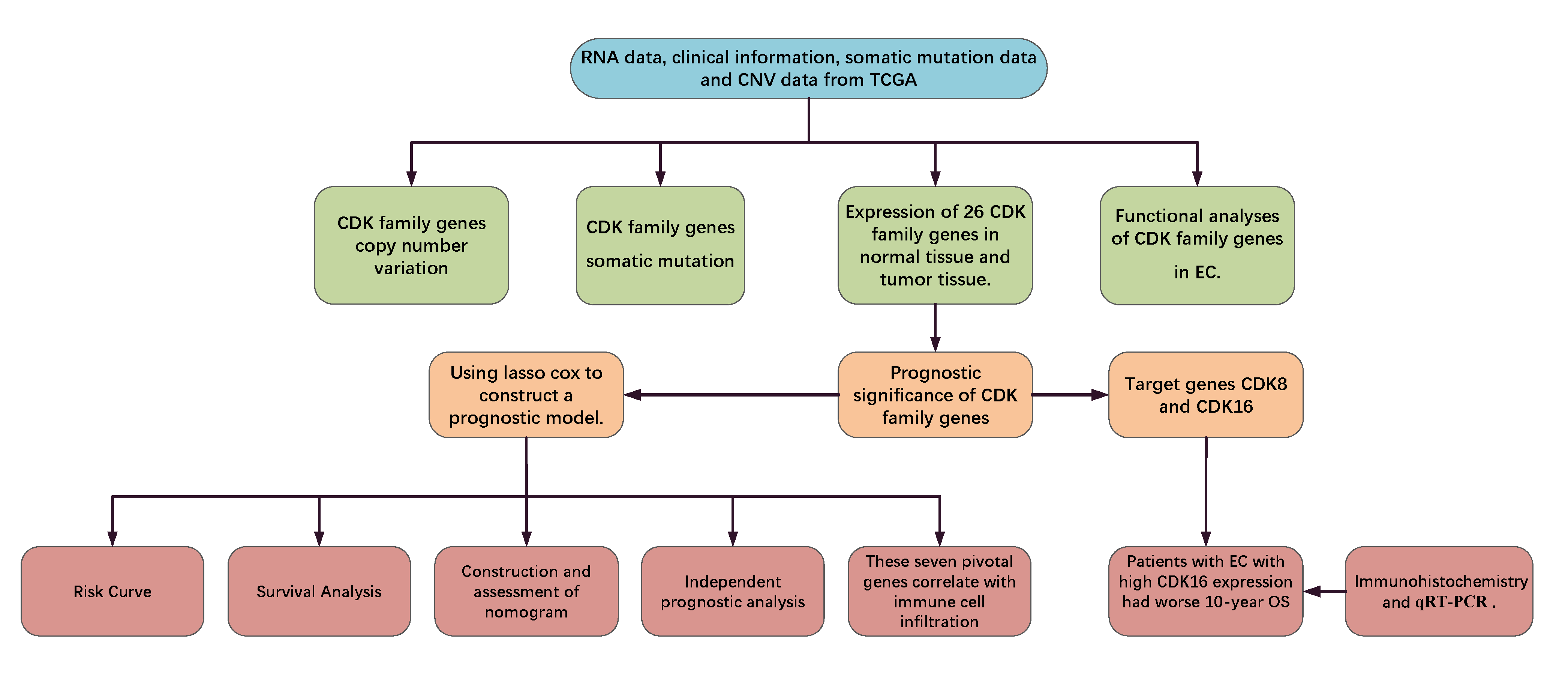

Supplement: Supplementary file 2 [file Image1.TIFF]
