# Supplementary material for: Endometrial cancer prognosis prediction using correlation models based on CDK family genes
Source: Front Genet. 2022 Oct 10;13:1021600. doi: 10.3389/fgene.2022.1021600 (PMC9589062; doi:10.3389/fgene.2022.1021600)
Supplement: Supplementary file 4 [file Table5.DOCX]

<https://www.jianguoyun.com/p/DX4NoKUQ--r7ChinjNoEIAA>
